# Leg Length Discrepancy Due to Loss of Femoral Antecurvatum After Elastic Stable Intramedullary Nailing of Diaphyseal Fractures of the Femur in Children

**DOI:** 10.7759/cureus.6343

**Published:** 2019-12-11

**Authors:** Panagiotis V Samelis, Eftychios Papagrigorakis, Theodore Troupis, Panagiotis Koulouvaris

**Affiliations:** 1 Orthopaedics, Children’s General Hospital Panagiotis & Aglaia Kyriakou, Athens, GRC; 2 Orthopaedics, KAT Trauma Hospital, Athens, GRC; 3 Surgery, University of Athens Medical School, Athens, GRC; 4 Orthopaedics, Attikon University Hospital, Athens, GRC

**Keywords:** children, elastic, intramedullary, femur, fracture, discrepancy, limb, length, antecurvatum, nail

## Abstract

Limb length discrepancy (LLD) is a frequent complication after elastic stable intramedullary nailing (ESIN) of femoral shaft fractures in children. It is the result of either shortening or lengthening of the affected limb. A shorter limb is usually observed when there is no strict adherence to the main indication of the technique, which is a transverse or short oblique fracture of the diaphysis. A longer limb may be the result of either improper reduction and significant dissociation of fracture fragments, or stimulation of growth of the fractured bone of the child, known as overgrowth. We describe a potential third cause of limb lengthening after treating femoral shaft fractures with the ESIN technique. LLD may be the result of acute femur lengthening due to the loss of normal femoral antecurvatum.

## Introduction

Elastic stable intramedullary nailing (ESIN) using a pair of titanium nails is a widely accepted method of treatment of long bone diaphyseal fractures in children, leading to fast recovery, short hospital stay, and fewer complications compared with other methods of treatment [1-4]. Opposite to typical rigid intramedullary nailing for fractures of the femur, ESIN enters the femoral diaphysis not through, but between the growth plates of the long bone, thus avoiding potential growth disturbance secondary to physis injury and avascular necrosis of the femoral head secondary of injury of the nutrient vessels of the femoral head [1,5]. Unfortunately, this is accomplished in expense of fracture stability and less control on the mechanical and torsional profile of the affected limb. Fortunately, the rapid healing and remodelling potential of the growing skeleton of the child compensates minor deviations from normal [1].

The main indication of ESIN is a transverse or short oblique long bone diaphyseal fracture of the growing skeleton in children between the ages of five and 12 years and weighing less than 50 kg [6]. Due to the simple operative technique and the rapid healing potential of the growing skeleton of the child, surgeons increasingly use this technique even in oblique or comminuted fractures [1,5,7].

The ESIN technique is quite simple; under image intensification, the nails are inserted from opposite sides of the distal femoral metaphysis, advanced towards the fracture, and anchored to the proximal femoral metaphysis after fracture reduction. Symmetric bending of the elastic nails prior to insertion is an integral part of this technique, in order, in terms of biomechanics, to obtain three-point fixation between each nail and the bone [8].

Complications such as skin problems due to prominent hardware, infection, fracture nonunion, hardware failure (bending, breakage) and complications during hardware removal may be observed in children treated with the ESIN technique [2,8]. The complications rate is higher in more complex fractures and in children weighing more than 40 kg [5,9].

Some complications are inherent with the ESIN technique. Until callus consolidation is complete, ESIN allows some movement (telescoping, bending, translation, torsion) at the fracture site. This may result in loss of longitudinal, torsional and angular alignment of the fracture and to subsequent malalignment and limb length discrepancy (LLD) [4-5].

The femoral shaft presents a normal anterior sagittal bow, termed antecurvatum. Femoral antecurvatum is the result of the intrinsic growing pattern and the extrinsic mechanical loading of the growing femur. It is present in the fetus and shows geographical and racial variance [10]. The normal radius of curvature of the femur is approximately 120 cm +/- 36 cm irrelevant of age [11]. Knowledge of the normal femoral antecurvatum is important in the typical rigid intramedullary nailing of femoral shaft fractures in adults, since the radius of curvature of the nail should correspond to the radius of anterior bowing of the femur, in order to avoid postoperative complications such as anterior femoral cortex penetration or hip and knee pain [10,11].

In children, anatomic reduction of femoral shaft fractures should include the restoration of femoral antecurvatum as well. Current literature lacks information about the effect of ESIN on femoral antecurvatum. In clinical practice, changes of femoral antecurvatum are rather ignored, unless they are extreme and cause significant discomfort to the patient. However, it seems that ESIN fails to restore femoral antecurvatum. Immediate postoperatively and as long as the patient is bedridden, the femur may assume a straight configuration on the sagittal plane (loss of antecurvatum). Loss of femoral antecurvatum results in a straight femoral shaft in the sagittal plane and may lead to acute postoperative leg lengthening after treatment of stable (transverse, short oblique) diaphyseal fractures with the ESIN technique. Prolonged bed stay may turn this finding to a permanent malunion and, along with potentially missed fracture diastasis and overgrowth, contribute to LLD with a longer affected limb.

The aim of this study is to describe the loss of femoral antecurvatum as a potential complication of the treatment of femoral shaft fractures with the ESIN technique that may lead to LLD due to acute lengthening of the operated femur.

## Case presentation

**Figure 1:**
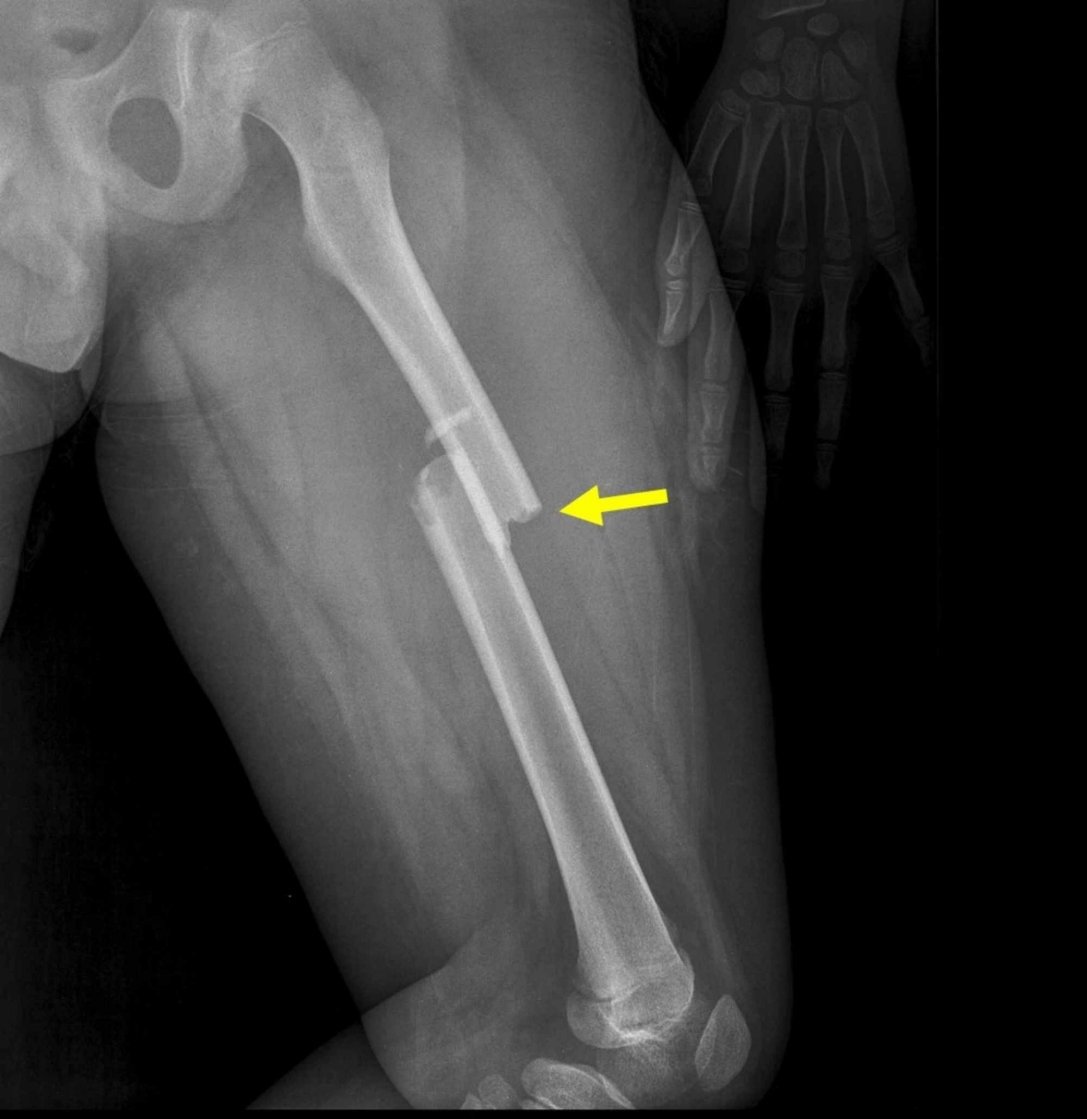
Midshaft transverse/short oblique fracture with minimal comminution of the diaphysis of the left femur of an 11-year-old boy Arrow indicates the fracture of the femur.

**Figure 2:**
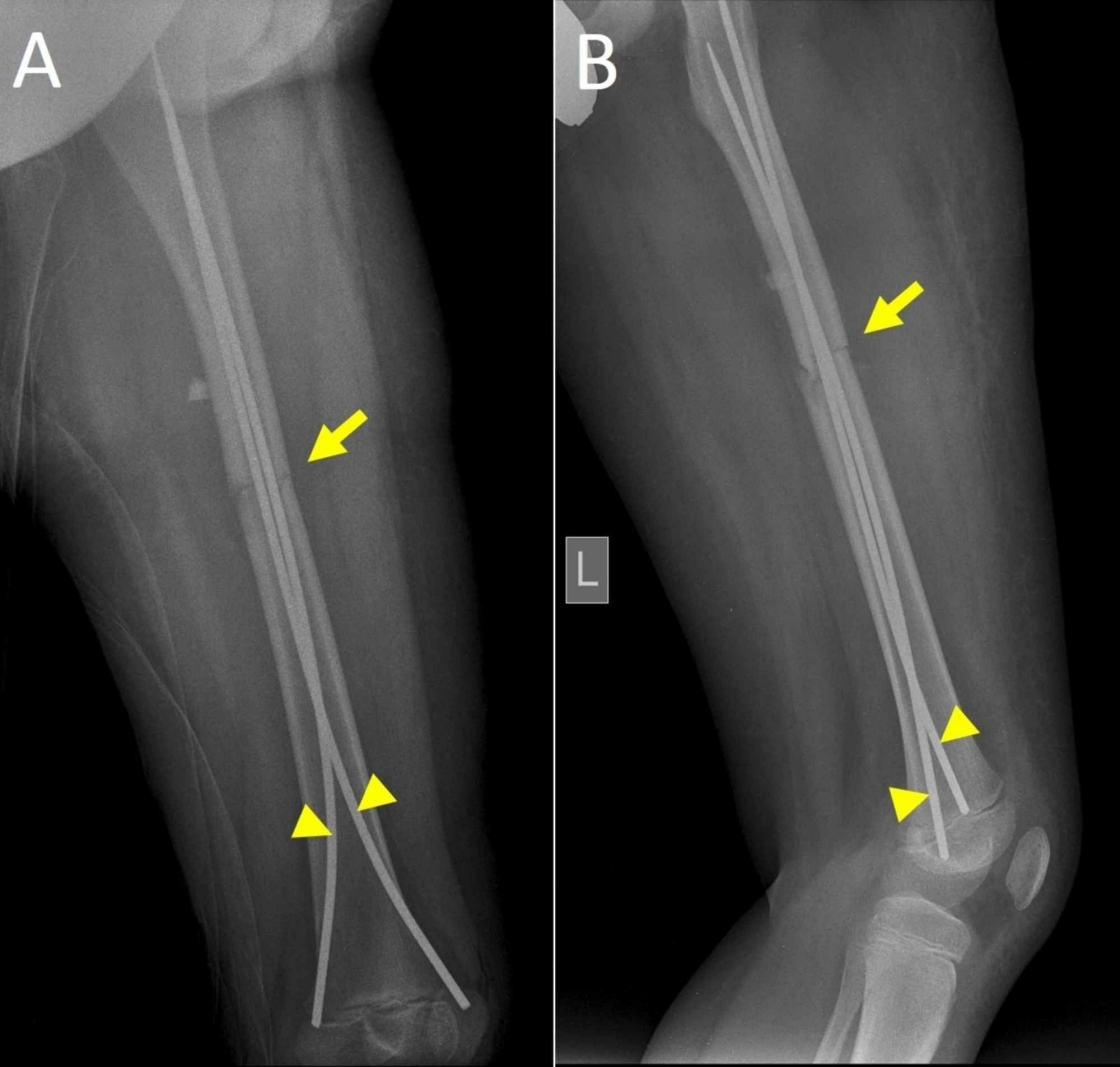
Postoperative X-ray after elastic stable intramedullary nailing (ESIN) of the left femur A. Anteroposterior view, B. Lateral view. Arrows indicate the fracture after reduction and osteosynthesis. Arrowheads indicate the titanium nails. Slight diastasis of the fracture fragments on both views is seen. Loss of femoral antecurvatum is evident on the lateral X-ray view.

An 11-year-old boy presented with a transverse fracture of his left femur after a fall from height (Figure 1). Surgical treatment using titanium nails according to the principles of the ESIN technique was decided. Traction table and image intensification were used. Nails of 3.5 mm width were chosen, corresponding approximately to 40% of the width of the narrowest diameter of the femoral shaft. Prior to insertion, the nails were bent three times the width of the medullary canal. The nails entered the medullary canal from opposite sides of the distal femoral metaphysis and were advanced retrogradely up to the fracture level. After closed fracture reduction, the nails were further advanced into the proximal fragment and anchored firmly into the proximal femoral metaphysis (Figure 2). Care was taken to obtain accurate reduction without a gap between the fracture fragments.

**Figure 3:**
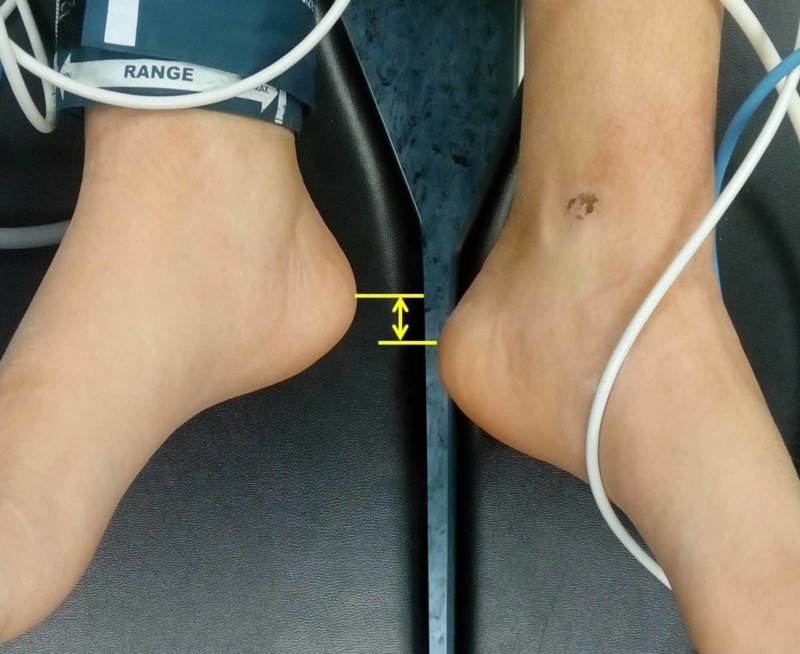
Immediate postoperative limb length discrepancy (LLD) The operated left leg is longer than the contralateral. Arrow indicates LLD of approximately 1.5 cm.

After resuscitation and transfer of the patient from the traction table to the stretcher, LLD due to a longer fractured limb was evident. The operated limb was about 1.5 cm longer than the contralateral (clinical measurement). LLD was evident as long as the patient was supine (Figure 3).

Follow up examinations focused on fracture healing and on LLD. Partial weight bearing as tolerated by pain was recommended. The patient adapted well to the initially longer limb.

**Figure 4:**
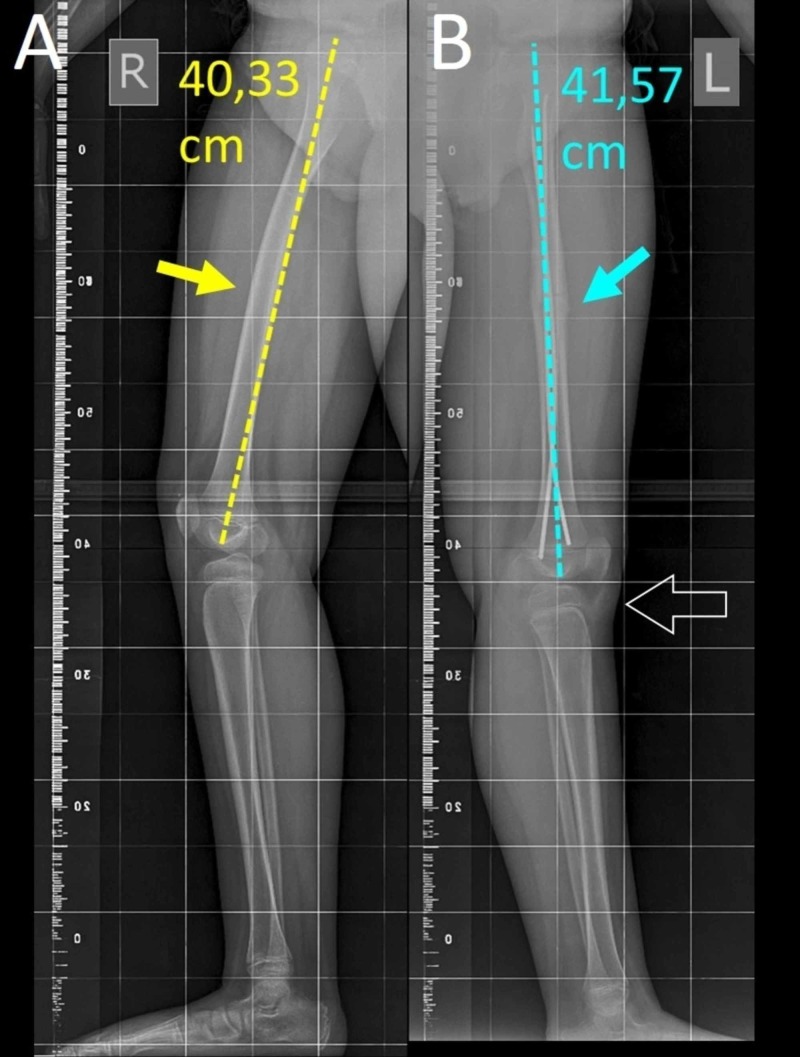
Long standing lateral X-ray view of the right (A) and the left (B) femur three months after surgery Limb length discrepancy with a longer left femur is obvious (+1.24 cm). A. Normal femoral antecurvatum of right femur (yellow arrow), B. The left femur is straightened due to loss of femoral antecurvatum (blue arrow). The left leg presents apparent (false) knee recurvatum secondary to loss of femoral antecurvatum (arrow outline).

The patient was dismissed one week postoperatively with instruction of partial weight bearing as tolerated for one month. Progressive weight bearing was recommended on follow-up examinations, according to radiologically confirmed callus formation. At three months postoperatively, fracture healing was complete and the patient was encouraged to resume noncompetitive sports. On clinical examination, slight ipsilateral knee recurvatum might be the only hint for the straightened femur. Three months after surgery, loss of femoral antecurvatum of the operated limb was still evident on the long limb standing lateral X-ray. LLD due to longer operated femur of +1.24 cm (radiological measurement as seen in Figure 4) persisted, compared to the contralateral femur. The ipsilateral tibia, was 2 mm longer than the contralateral (radiological measurement); however, this finding was considered as non-significant for the total LLD.

**Figure 5:**
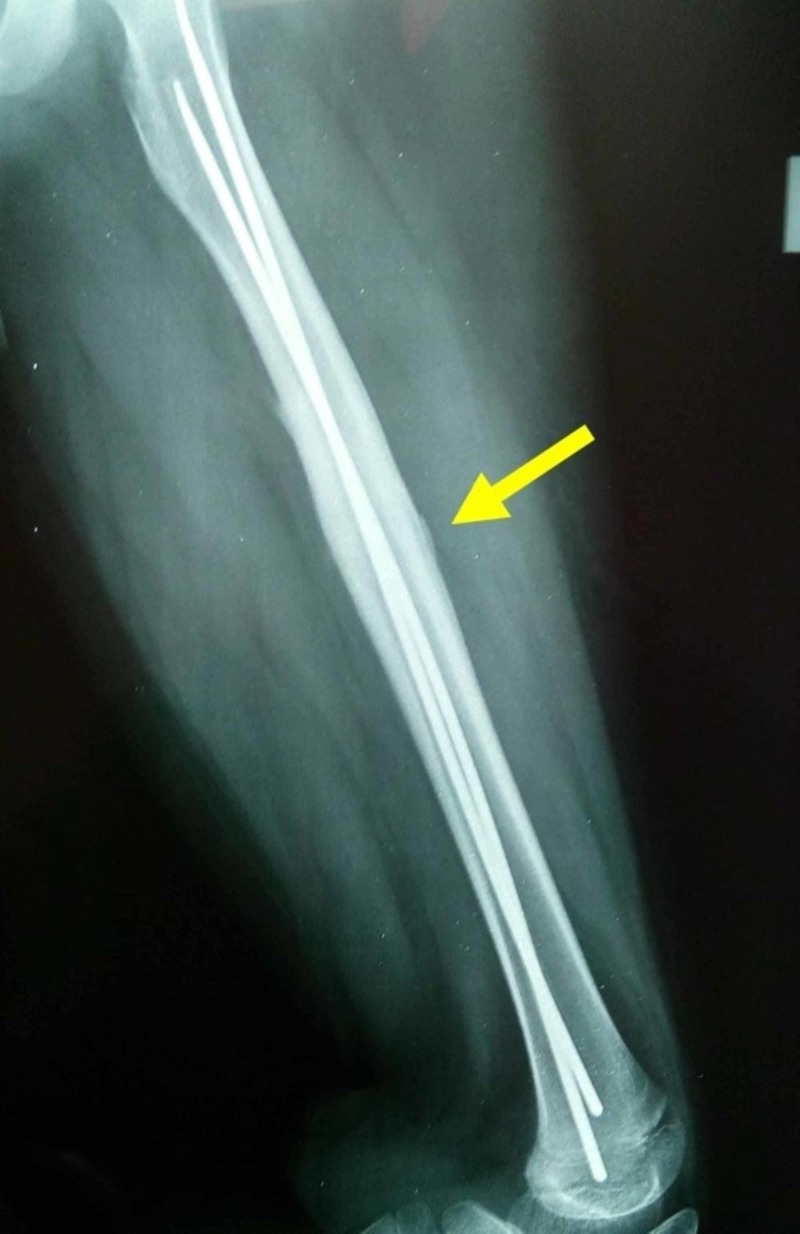
Lateral X-ray view of the operated left femur six months after surgery; fracture healing is complete Arrow indicates complete fracture healing and a partially restored antecurvatum of the left femur.

Healing was complete at six months postoperatively. The patient was asymptomatic. Walking and participation in sports was unrestricted. Hardware removal was scheduled. A slight LLD of less than 5 mm was detected on clinical examination. Femoral antecurvatum was partially restored (Figure 5).

## Discussion

There are two types of LLD after ESIN of femoral shaft fractures in children:

The first type is limb shortening, seen with unstable fracture patterns (comminuted, spiral). Shortening of the fractured limb may be the immediate result of surgery (missed during surgery) or may be due to postoperative loss of reduction (telescoping of fracture fragments due to unstable osteosynthesis) [5]. Limb shortening after a diaphyseal long bone fracture is never attributed to growth deceleration of the injured bone and contrary to overgrowth, is always considered a complication.

The second type of LLD is a longer affected limb, that may be observed either immediately after surgery (early, acute lengthening) or may be a late result, known as overgrowth.

First described by Truesdell in 1921, overgrowth was attributed to stimulation of growth secondary to trauma. Most authors report an average overgrowth of 8-10 mm after femoral shaft fracture [12-14]. Overgrowth is not confined to the fractured femur, but, to a lesser extent, involves the non-fractured ipsilateral tibia as well [10-11]. It seems that acceleration of growth reaches its peak at three months postinjury and returns to normal after 40 to 60 months for tibial and femoral fractures respectively [11]. Other authors confine overgrowth within the healing period and support that the difference at the end of healing is permanent. It has been postulated that it is not necessary to strive for perfection of reduction of long bone fractures in children, because overgrowth and remodeling compensate well for minor deviations from normal [11]. Epiphysiodesis, in order to address overgrowth greater than 2 cm, is rarely necessary [1,3].

**Figure 6:**
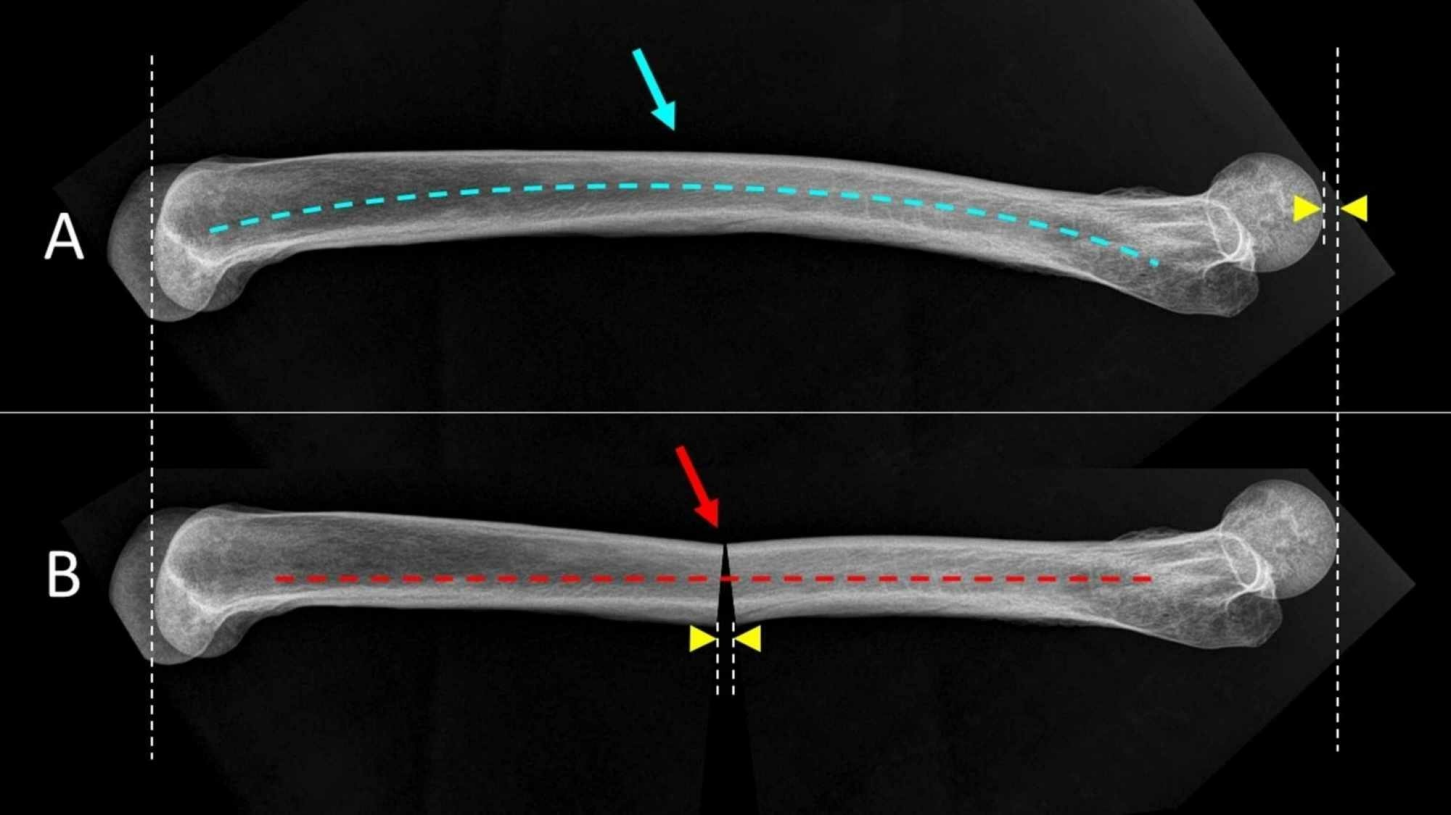
The effect of femoral antecurvatum on the length of the femur is shown on the lateral X-ray of a femoral bone A. Normal femoral antecurvatum. B. Loss of femoral antecurvatum after ESIN A. Blue arrow indicates normal femoral antecurvatum (blue dashed line), B. Red arrow indicates virtual transverse midshaft femoral fracture. Osteosynthesis with ESIN (red dashed line) leads to loss of the anterior curvature of the femur, either because the titanium nails are straight on the sagittal plane or secondary to the inherent sagittal instability of the bone-nail construct when treated with the ESIN technique. As a result, the femur becomes relatively longer than prior to the fracture (arrowheads). ESIN: elastic stable intramedullary nailing.

Acute lengthening after a femoral shaft fracture treated with ESIN has two aspects. One aspect is fracture diastasis, that is, a gap is present between the main fracture fragments, that was missed and left uncorrected during surgery. However, acute lengthening may be the result of a previously not described complication of femoral shaft fractures in children, treated especially with the ESIN technique. This complication is not a late effect, but is rather an adverse effect of the ESIN technique on the normal femoral antecurvatum. The concept of how a change of the femoral antecurvatum after a midshaft fracture affects femoral length is shown in Figure 6.

In fact, the ESIN technique inherently fails to restore femoral antecurvatum after a femoral shaft fracture. This is because preoperative bending of the nails applies only in the frontal plane, thus providing some resistance only against varus-valgus angulation. On the contrary, the nails are straight in the sagittal plane and hence, either force the diaphysis to assume a straight configuration too (the femur is "stretched" by the nail) or fail to provide fracture stability against anteroposterior angulation. Thus, the femur-nail construct sags in the sagittal plane under the weight of the affected leg as long as the patient is bedridden. 

Most studies consider deformities in the sagittal plane after ESIN of femoral shaft fractures (procurvatum, recurvatum) as late complications or malunions [5,15-16]. Few studies report on the immediate postoperative effect of ESIN on femoral antecurvatum. One study recommends the insertion of a third titanium nail in order to reinforce the bone-implant construct against deformation in the sagittal and frontal plane [5]. Other studies support that femoral antecurvatum is either spontaneously restored at three weeks after surgery or is not significantly affected by ESIN [1,8]. However, immediate postoperative (early) loss of femoral antecurvatum after ESIN of femoral shaft fractures is obvious on radiographs presented in several studies, but this finding is not commented on [17-19]. One of these studies shows the X-rays of a patient where it is evident that the immediate postoperative loss of femoral antecurvatum remains unchanged at one and 10 months postoperatively [19]. 

Interestingly, some studies report that transverse femoral fractures in children, treated using ESIN, have the highest tendency for posttraumatic lengthening compared with comminuted or spiral fractures [1,4]. This is consistent with the presented case and at least some part of the LLD could be attributed not only to overgrowth or to a postoperative fracture gap, but also to potentially decreased femoral antecurvatum after ESIN of transverse femoral shaft fractures.

Gulati et al. did not find significant impact of sagittal angulation of the femur on the final limb length after conservative treatment of femoral shaft fractures in children younger than 10 years of age [20]. To our knowledge, most studies that deal with LLD after femur fractures, treated with the ESIN technique, do not take into account the potential impact of sagittal angulation of the femur on final limb length. Thus, the validity of published data about overgrowth and LLD after a femoral shaft fracture treated with the ESIN technique is questioned, since the length of the femur after a fracture depends not only on overgrowth, but also on the adverse effect of ESIN on normal femoral antecurvatum.

## Conclusions

Immediate postoperative limb lengthening secondary to loss of femoral antecurvatum is a benign and self-limiting complication after ESIN of femoral shaft fractures in children. This complication is inherent with the ESIN technique due to either the straight configuration of the titanium nails in the sagittal plane or posterior sag of the bone-nail construct. In the presented case, loss of femoral antecurvatum was almost corrected at hardware removal and the patient had an excellent outcome. Immediate postoperative loss of femoral antecurvatum may go undiagnosed in bilateral femoral fractures, as in polytrauma patients. Early ambulation and controlled weight bearing according to callus formation is strongly indicated, especially in stable fracture types of the femoral diaphysis (transverse, short oblique fractures), in order to progressively restore femoral antecurvatum and to avoid permanent lengthening of the affected femur, resulting in potentially significant limb length discrepancy.
